# Macrophage Mannose Receptor CD206 Predicts Prognosis in Community-acquired Pneumonia

**DOI:** 10.1038/s41598-019-55289-2

**Published:** 2019-12-10

**Authors:** Tsuchiya Kazuo, Yuzo Suzuki, Katsuhiro Yoshimura, Hideki Yasui, Masato Karayama, Hironao Hozumi, Kazuki Furuhashi, Noriyuki Enomoto, Tomoyuki Fujisawa, Yutaro Nakamura, Naoki Inui, Koushi Yokomura, Takafumi Suda

**Affiliations:** 1 Second Division, Department of Internal Medicine, Hamamatsu University School of Medicine, Hamamatsu, Japan; 2 Department of Respiratory Medicine, Seirei Mikatahara Hospital, Hamamatsu, Japan

**Keywords:** Prognostic markers, Pattern recognition receptors, Infection

## Abstract

CD206, a mannose receptor, is mainly expressed on the surface of alternatively activated macrophages where it acts as a pattern recognition receptor and plays a role in innate and adaptive immunity. This study investigated serum soluble CD206 (sCD206) levels in community-acquired pneumonia (CAP) and examined their clinical significance. sCD206 concentrations were measured in the sera of two independent cohorts with CAP (127 and 125 patients, respectively) and 42 controls. The expression of CD206 in the lung from autopsied cases was also examined. Patients with CAP showed significantly elevated sCD206 levels than did the controls (p < 0.0001). Notably, fatal CAP patients had more than two-fold higher sCD206 concentrations than survivors in both cohorts (p < 0.0001). Serum sCD206 concentrations were associated with Pneumonia Severity Index (PSI) and CURB-65 values. Importantly, even fatal CAP patients classified as PSI I-IV, CURB65 0–2 or age <75 years had comparatively higher levels of sCD206 than those classified as PSI V, CURB-65 3–5 or age ≥75 years. Immunohistochemically, the infiltration of CD206^+^ macrophages was found in the lungs of fatal cases. Elevated levels of sCD206 are associated with CAP prognosis, suggesting sCD206 might be a potential biomarker to predict severity for CAP.

## Introduction

Community-acquired pneumonia (CAP) is the most common infectious disease and a leading cause of morbidity and mortality worldwide^1^. The pathogenesis of infectious disease varies based on the causative agent involved, as well as host immune status, which in turn, is regulated via a complex mechanism. Inflammation is an essential defense mechanism at the mucosal level, wherein immune cells are activated in response to invading pathogens. At the same time, immunosuppression or immune tolerance protects the host from excessive or inappropriate immune activation. Macrophages have a pivotal role in the innate immune response, and phenotypically-polarized macrophages are generally classified into two major subtypes, namely pro-inflammatory M1 and anti-inflammatory M2 macrophages^2,3^. We previously demonstrated that soluble CD163 and indoleamine 2,3-dioxygenase (IDO), an immunoregulator produced by macrophages and dendritic cells, are associated with disease severity and the prognosis of CAP^4,5^. This suggests that immunomodulatory molecules can potentially serve as surrogate markers for infectious diseases.

CD206 is a mannose receptor expressed on alternatively activated macrophages, termed M2 macrophages. M2 macrophages have an anti-inflammatory phenotype to resolve excess inflammation, promoting wound healing and also inducing immunotolerance^2,3^. On the surface of macrophages, CD206 acts as a pattern recognition receptor (PPR) for various pathogens including viruses, fungi, and bacteria; further, this molecule recognizes their mannan-coated cell walls or envelopes, and functions following endocytosis, phagocytosis and antigen presentation^6–9^. Additionally, polysaccharide from *Streptococcus pneumoniae* and lipopolysaccharide from *Klebsiella pneumoniae* bind CD206^10^, indicating roles for these molecules in infectious disease. Upon proteolytic cleavage of the membrane-bound form, the soluble form of CD206 (sCD206) is produced. Importantly sCD206 also recognizes sulphated and mannosylated carbohydrates^10,11^. As this molecule can be identified at peripheral sites, we hypothesized that evaluating its levels might represent macrophage activity and could be a potential biomarker for CAP. Therefore, in this study, we measured sCD206 levels in patients with CAP and evaluated their clinical implications. We also examined membrane-bound CD206 in the lungs of autopsied cases by immunohistochemistry.

## Results

### Clinical characteristics

Clinical characteristics of 127 patients with CAP in Cohort1 and 125 patients in Cohort2 are summarized in Table 1. There were no obvious differences in sex, but Cohort2 comprised older individuals than Cohort1. The presence of respiratory failure (SaO_2_ < 90% or PaO_2_ < 60 Torr) or impaired consciousness was not differed between the cohorts. Most patients showed elevated levels of C-reactive protein (CRP) and increased white blood cell counts. Serum levels of procalcitonin (PCT) were measured for 78 patients in Cohort2, of which 45 showed positive PCT (cut-off, 0.5 ng/ml). Blood cultures were positive for samples from eight patients (6.3%) in Cohort1 and seven patients (5.6%) in Cohort2.

**Table 1:** Clinical characteristics of patients with community acquired pneumonia.

	Cohort1 (n = 127)	Cohort2 (n = 125)	*p*-value
Sex, M/F	77/50	77/48	0.898
Age, years	73.0 (21–99)	76.0 (31–93)	0.006
**Comorbidities**			
Congestive heart failure (n, %)	23 (18.1)	15 (12.0)	0.218
Chronic pulmonary disease (n, %)	47 (37.3)	66 (52.8)	0.016
Renal disease (n, %)	4 (3.1)	17 (13.6)	0.003
Diabetes mellitus (n, %)	11 (8.7)	33 (26.4)	<0.001
Chronic liver disease (n, %)	5 (3.9)	11 (8.8)	0.128
Cerebrovascular disease (n, %)	23 (18.1)	11 (8.8)	0.042
Neoplastic disease (n, %)	12 (9.4)	38 (30.4)	<0.001
Immunosuppressive agents (n, %)	7 (5.6)	33 (26.4)	<0.001
**Clinical characteristics**			
Body temperature,	37.8 (35.0–40.8)	37.8 (35.6–40.3)	0.462
Systolic blood pressure <90 mmHg (n, %)	7 (5.5)	7 (5.6)	1.000
Confusion (n, %)	24 (19.0)	12 (9.6)	0.047
Respiratory Failure (SaO_2_ < 90%) (n, %)	64 (50.8)	74 (59.2)	0.205
PaO_2_/FiO_2_ ratio	291.00 (49.8–428.60)	260.95 (42.1–496.43)	0.006
**Laboratory findings**			
BUN (mg/dL)	16.70 (4.5–206.0)	20.00 (7.3–172.5)	0.008
Cre (mg/dl)	0.75 (0.23–6.69)	0.87 (0.38–10.60)	0.003
Alb (g/dl)	3.50 (2.30–5.00)	3.10 (1.80–4.50)	<0.001
WBC (/μL)	10760 (2300–37000)	10170 (1000–29330)	0.932
CRP (mg/dl)	12.00 (0.17–57.65)	12.04 (0.09–50.17)	0.886
PCT (ng/ml)	ND	0.68 (0.02–87.8)	NA
Bacteremia	8 (6.3)	7 (5.6)	0.136

BUN; blood urea nitrogen, Cre; creatinine, Alb; albumin, CRP; C-reactive protein, PCT; procalcitonin, P/F ratio; PaO_2_/FiO_2_ ratio.

Data are shown by median (minimum-maximum).

The severity and outcomes of CAP patients are presented in Table 2. The median Pneumonia Severe Index (PSI) was 91 points in Cohort1 and 107 points in Cohort2, respectively, indicating that severity tended to be higher in Cohort2 (p = 0.022), and more than 50% of patients were categorized in PSI class ≥IV. The median CURB-65 score was 1.0 in each cohort, and this score indicated higher severity in Cohort2 (p = 0.032); approximately half of patients were classified as CURB-65 Class 0–1. The proportions of CAP patients requiring mechanical ventilation or ICU administration were not different between the cohorts. Overall mortality due to respiratory failure or multiple organ failure associated with CAP after admission was 10.2% (13 patients) in Cohort1 and 7.2% (nine patients) in Cohort2 but did not differ between the cohorts. The identified pathogens were showed in the Supplement Table 1.

**Table 2:** Severity and Outcome of patients with community acquired pneumonia.

	Cohort1 (n = 134)	Cohort2 (n = 125)	*p*-value
**PSI, no (%)**			
I	15 (11.8)	3 (2.4)	0.022
II	18 (14.2)	13 (10.4)	
III	30 (23.6)	27 (21.6)	
IV	40 (31.5)	51 (40.8)	
V	24 (18.9)	31 (24.8)	
**CURB65, no. (%)**			
0–1	71 (55.9)	65 (52.0)	0.032
2	27 (21.3)	43 (34.4)	
3–5	29 (22.8)	17 (13.6)	
IPPV (n, %)	11 (8.7)	5 (4.0)	0.195
NPPV (n, %)	5 (3.9)	8 (6.4)	0.409
ICU admission (n, %)	12 (9.4)	9 (7.2)	0.650
Duration of hospitality	14.5 (0–149)	15 (2–75)	0.189
Mortality (n, %)	13 (10.2)	9 (7.2)	0.504

IPPV; invasive positive pressure ventilation, NPPV; non-invasive positive pressure ventilation, ICU; intensive care unit.

Data are shown by median (minimam-maximam).

### Serum concentrations of sCD206 in Cohort1

**Figure 1:**
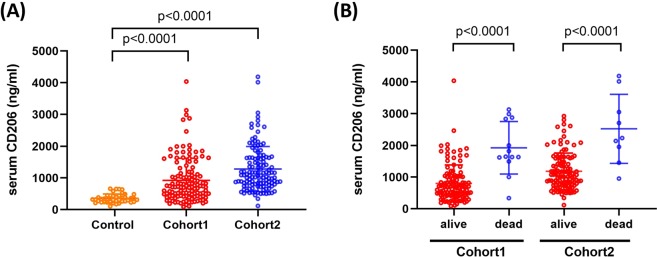
Serum concentrations of sCD206 in patients with community-acquired pneumonia (CAP). Serum concentrations of sCD206 in patients with CAP and control subjects (**A**), and in CAP patients with non-survivors and survivors (**B**). *P*-values were determined by Mann-Whitney U test.

The serum concentrations of sCD206 in CAP patients in Cohort1 are presented in Fig. 1. The mean serum level of sCD206 in patients with CAP was 718.8 ng/ml [90.38–4035.55], whereas that in healthy controls was 329.2 ng/ml [104.87–660.92], indicating significantly elevated levels in patients with CAP (p < 0.0001, Fig. 1A). Further, the patients who died had significantly higher levels of serum sCD206 (1644 ng/ml [340.41–3130.15]) compared to those in survivors (683 ng/ml [90.38–4035.55], p < 0.0001; Fig. 1B).

### Serum concentrations of sCD206 in Cohort2

We separately evaluated sCD206 levels in patients with CAP in another cohort. In Cohort2, similar to that in Cohort1, patients with CAP had significantly higher levels of sCD206 than controls (1086 ng/ml [118.155–4188.05] vs 329.2 ng/ml [104.87–660.92], p < 0.0001, Fig. 1A). Additionally, CAP patients who died had more than two-fold higher levels of sCD206 compared to those in surviving CAP patients (2232 ng/ml [954.35–4188.05] vs. 1058 ng/ml [118.155–2923.375], p < 0.0001; Fig. 1B**)**.

#### Correlations between sCD206 and disease severity or clinical parameters

**Figure 2:**
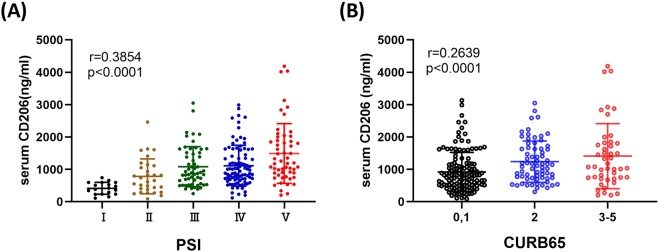
Serum concentrations of sCD206 in patients with community-acquired pneumonia (CAP) according to the disease severity. Serum concentrations of sCD206 in patients with CAP according to the PSI (**A**) and CURB65 (**B**). *P*-values were determined using the ANOVA test.

We next evaluated serum sCD206 levels according to CAP severity using PSI and CURB-65 scales. As shown in Fig. 2A,B, serum concentrations of sCD206 were increased according to severity of the CAP index. Additionally, correlation analyses showed that sCD206 was positively correlated with PSI score and CUB-65 score, as well as BUN and CRP levels, but was negatively associated with PaO_2_/FiO_2_ ratio (P/F ratio) and albumin levels (Supplement Table 2).

#### sCD206 levels in patients and non-survivors according to disease severity and age

**Figure 3:**
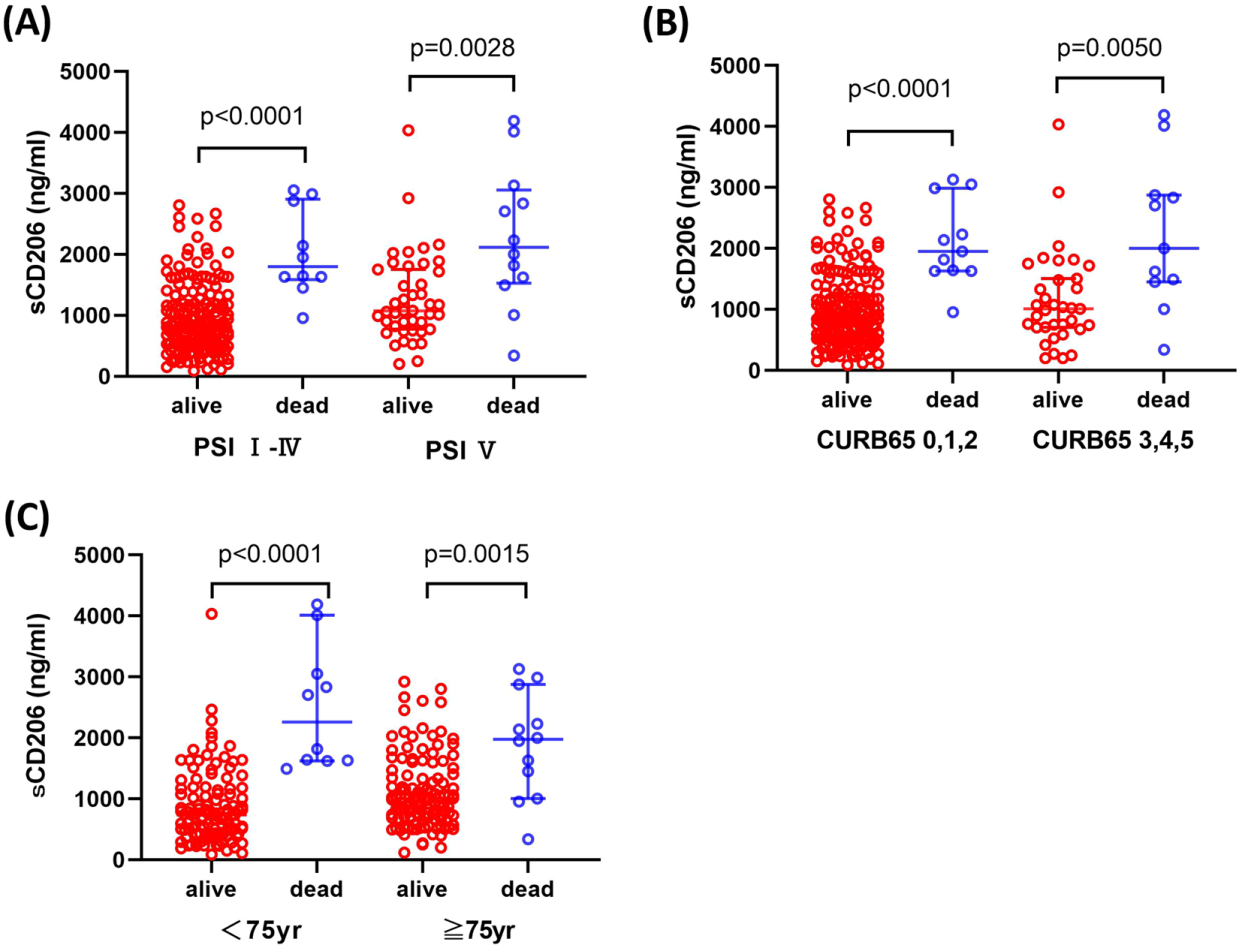
Serum concentrations of sCD206 in patients with community-acquired pneumonia (CAP) according to prognosis. Serum concentrations of sCD206 in patients with CAP according to prognosis classified by the PSI (**A**), CURB65 (**B**), and Age (**C**). *P*-values were determined by Mann-Whitney U test.

Although a significantly higher mortality rate was found for CAP patients classified into PSI V (p = 0.001), CURB-65 3–5 (p = 0.001) and age ≥75 years groups, more than 40% of the non-surviving patients were classified as PSI < V (n = 10, 45.5%), CURB-65 < 3–5 (n = 11, 50%), and age <75 years (n = 10, 45.5%). Thus, we next evaluated sCD206 levels in 22 patients compared to those in non-survivors. The patients with CAP who died had significantly elevated serum sCD206 levels than those in surviving patients regardless of severity in terms of PSI index (816.4 ng/ml [90.38–2806.85] vs. 1798.04 ng/ml [954.35–3050.13] for PSI I-IV as compared to 1072.62 ng/ml [200.64–4035.55] vs. 2116.15 ng/ml [340.41–4188.05] for PSI V; p < 0.0001 and p = 0.0028, respectively, Fig. 3A), and CURB65 index (835.85 ng/ml [90.38–2806.85] vs. 1951.77 ng/ml [954.35–3130.15] for PSI I-IV as compared to 1010.1 ng/ml [200.64–4035.55] vs. 2000.06 ng/ml [340.41–4188.05] for PSI V; p < 0.0001 and p = 0.0050, respectively, Fig. 3B). The patients with CAP who died had significantly elevated serum sCD206 levels in both age groups (741.15 ng/ml [90.38–4035.55] vs. 2261.3 ng/ml [1493.9–4188.05] for age <75 years as compared to 1005.81 ng/ml [118.155–2923.375] vs. 1975.92 ng/ml [340.41–3130.15] for age ≥75 years; p < 0.0001 and p = 0.0015, respectively, Fig. 3C). Additionally, when comparing non-survivors, the levels of sCD206 were comparable between PSI I-IV and PSI V, CURB65 0–2 and CURB65 3–5, and age <75 years and age ≥75 years.

### Univariate and multivariate analyses of prognostic factors

**Figure 4:**
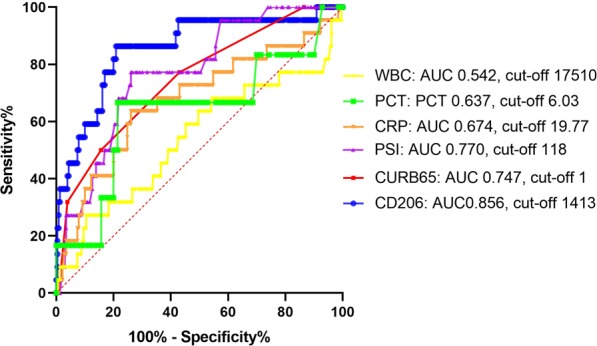
Receiver operator curve analysis for predicting mortality in patients with community-acquired pneumonia (CAP). Receiver operator curve analysis for predicting mortality in patients with CAP.

**Figure 5:**
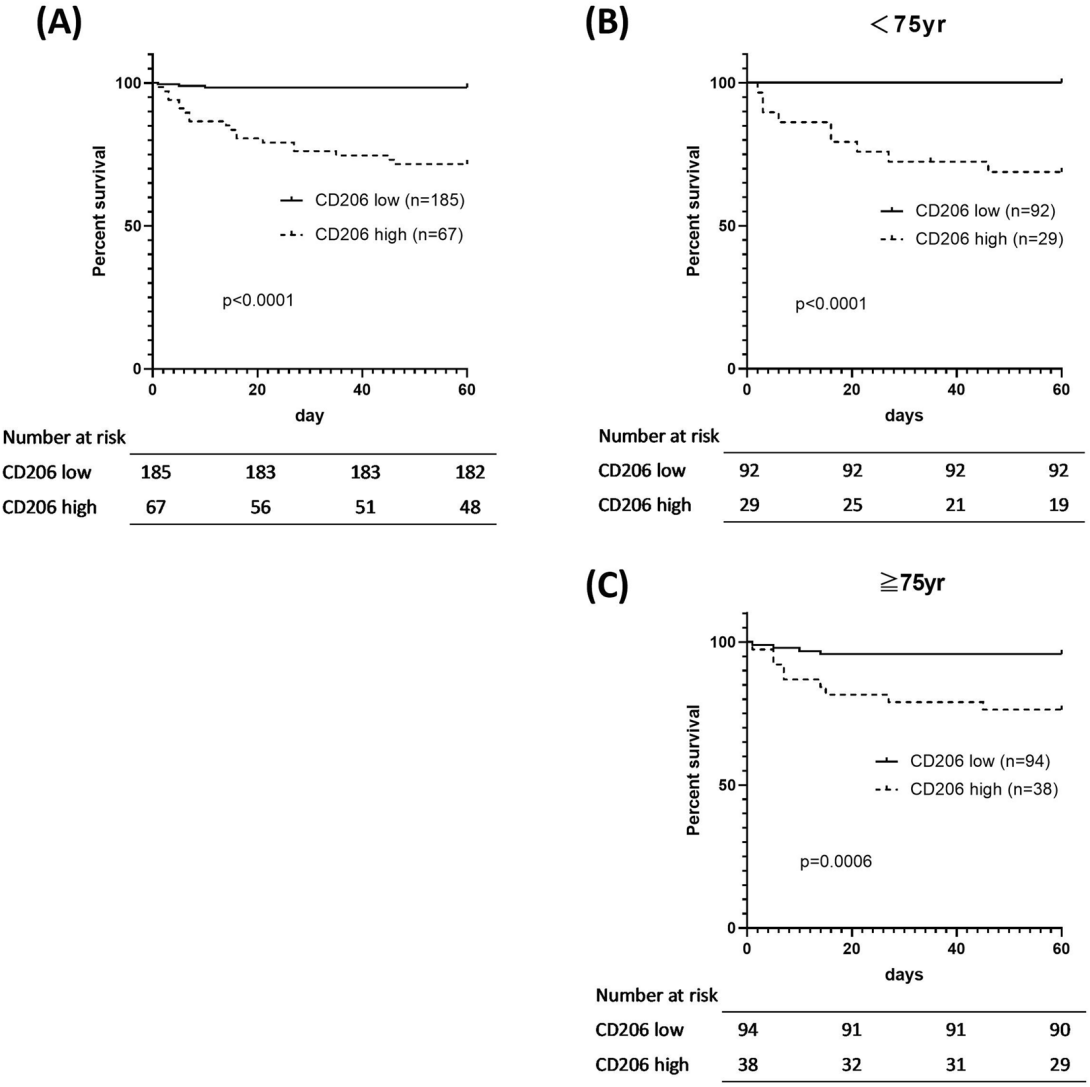
Kaplan-Meier curves of patients with community-acquired pneumonia (CAP). Kaplan-Meier curves of patients with CAP produced according to serum sCD206 concentrations. Kaplan-Meier curves of patients with CAP (**A**), CAP age <75 yr (**B**) and ≥75 yr (**C**) according to serum sCD206 concentrations. P-values were determined by the log-rank test.

We next calculated the optimal cut-off values for sCD206 to predict death using ROC analysis. Based on the ROC analysis, the area under the ROC curve (AUC) was 0.856 for sCD206 (Fig. 4), which was superior to that for CRP, PSI, or CURB65 score (0.674, 0.770, and 0.747, respectively). The AUC for PCT was 0.603 for 78 patient samples. Using the optimal cut-off for serum sCD206 levels (1413 ng/ml), sensitivity and specificity values obtained were 86.4% and 79.1%, respectively, with a likelihood ratio of 4.13. We then assessed the prognosis of patients with CAP based on this cut-off value, using the Kaplan–Meier method and log-rank test. The high-sCD206 (n = 67) group showed significantly lower survival rates than the low-sCD206 (n = 185) group (p < 0.0001; Fig. 5A). Although age-related scoring systems such as PSI and CURB65 are widely validated and confirmed^12,13^, certain situations have been associated with concerns about validity. For example, there are potential risks of overestimating severity in elderly patients and misclassifying younger patients with severe disease into lower classes^14–16^. Therefore, we performed a subgroup analysis of sCD206 according to patient age and confirmed that this cut-off worked, regardless of patient age (Fig. 5B,C). Finally, to determine the prognostic value of sCD206 with regard to outcome, we performed logistic regression analyses (Table 3). By univariate analysis and age/sex-adjusted multivariate analyses, sCD206, as well as PSI, CURB-65, and CRP, was found to be a significant predictor of death.

**Table 3:** Prediction of mortality: Logistic regression analysis.

Predictor	HR	95% CI	p-value	HR	95% CI	*p*-value
	Univariate analysis			Age and Gender adjusted Multivariate analysis		
Sex, M/F	1.12	0.454–2.790	0.7990			
Age, years	1.02	0.987–1.060	0.2060			
PSI (score)	1.030	1.010–1.0400	<0.0001	1.030	1.010–1.040	<0.0001
CURB65 (score)	2.31	1.560–3.430	<0.0001	2.310	1.540–3.460	<0.0001
Confusion	4.10	1.580–10.70	0.0038	3.930	1.500–10.30	0.0053
Bacteremia	8.93	2.650–30.20	0.0004	10.20	2.81–36.90	0.0004
BUN	1.02	1.010–1.040	0.0071	1.020	1.000–1.040	0.0108
Cre	1.34	1.030–1.75	0.0318	1.350	1.030–1.770	0.0311
Alb	0.24	0.103–0.56	0.0010	0.237	0.100–0.565	0.0012
CRP	1.06	1.020–1.100	0.0031	1.060	1.020–1.100	0.0032
P/F ratio	0.994	0.990–0.998	0.0027	0.994	0.990–0.998	0.0034
CD206 (/100 ng/ml)	1.190	1.120–1.270	<0.0001	1.190	1.120–1.270	<0.0001

Prediction of mortality with community-acquired pneumonia patients: univariate and multivariate analyses. Multivariate analysis was adjusted by sex and age. HR; hazard ratio, CI; confidence interval, BUN; blood urea nitrogen, Cre; creatinine, Alb; albumin, CRP; C-reactive protein, P/F ratio; PaO_2_/FiO_2_ ratio.

### Expression of CD206 in the lung biopsies of patients with CAP

**Figure 6:**
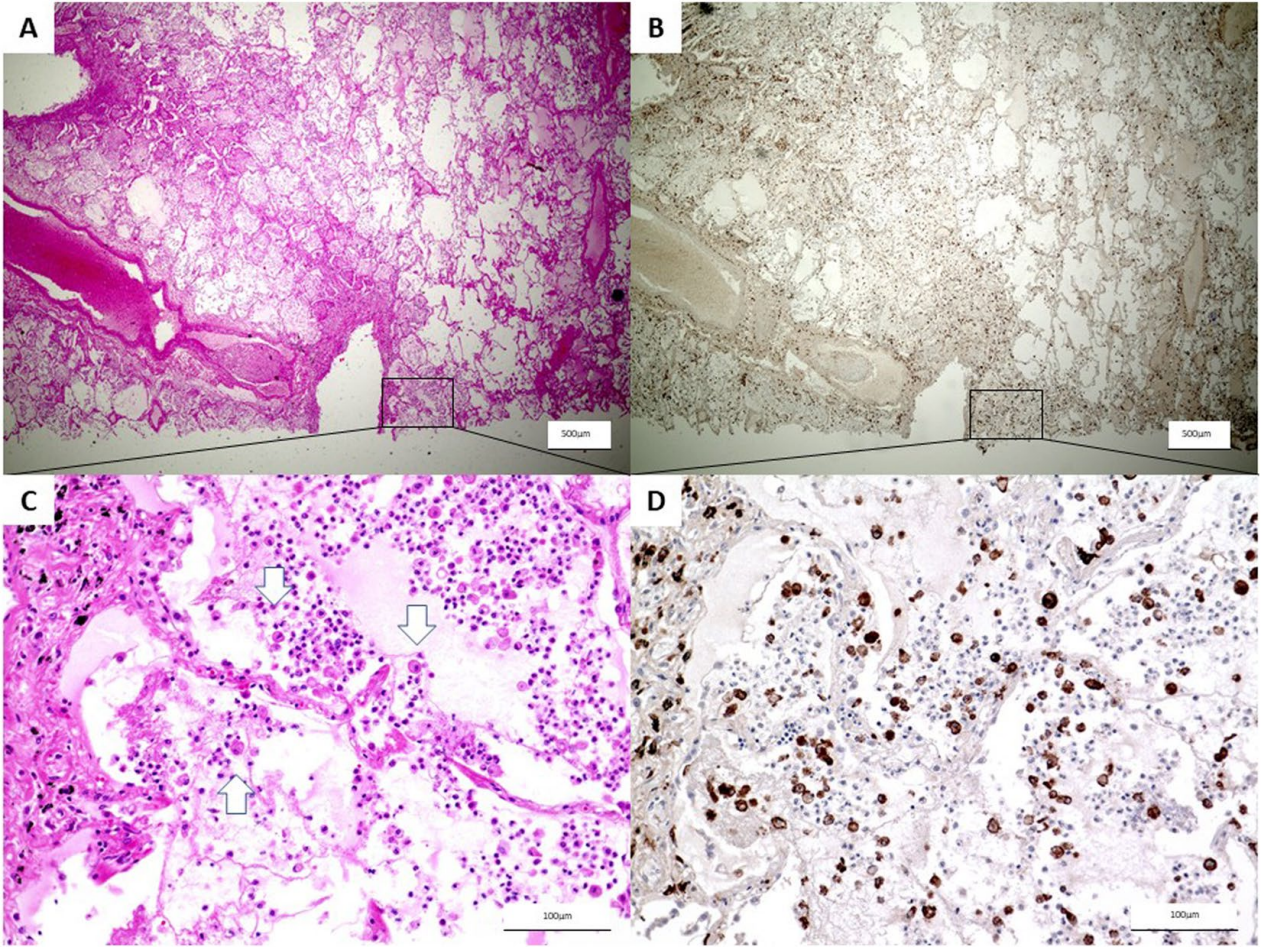
Immunohistochemical staining of CD206 in autopsied lungs of community-acquired pneumonia. 75-year-old male autopsy case, patient was dead in two days from the onset of pneumonia. (**A** ×20, **C** ×200) HE stain shows strong infiltration of inflammatory cells and macrophages (arrow) and pulmonary congestion in alveolar. (**B** ×20, **D** ×200) CD206 positive macrophages were observed in immunohistochemical staining.

To investigate the involvement of M2 macrophages and sources of sCD206 in patients with severe CAP, we assessed CD206 expression in autopsied-lung tissues from fatal CAP patients by immunohistochemistry. An autopsied lung specimen from a 75-year-old male with fatal CAP showed dense infiltration of neutrophils and CD206^+^ macrophages with fibrinous exudates in the alveolar space (Fig. 6). Similarly, the accumulation of CD206-positive macrophages was found in the lungs of the remaining cases (Supplement Figs. 1, 2). Meanwhile, only a few CD206-positive macrophages were found in the alveolar spaces in the resected lung sections from patient with early lung cancer (Supplement Fig. 3).

## Discussion

In the present study, we measured the serum sCD206 levels in patients with CAP and evaluated their clinical implications using two independent cohorts. Compared with control subjects, we found that patients with CAP demonstrated a significant increase in serum sCD206 that is associated with disease severity and prognosis. The accumulation of CD206^+^ macrophages in the alveolar and interstitial spaces was also found in the lungs of autopsied cases. Importantly, we found that sCD206 is a potential surrogate marker to predict CAP outcome.

Macrophages are abundant cells in the lungs and have pivotal roles in first-line defense and the maintenance of homeostasis^8,17^. On the surface of macrophages, CD206 serves as a PPR and functions in pathogen recognition and internalization by binding and scavenging unwanted glycoprotein and glycolipids^10,11^. Notably, CD206 itself can bind polysaccharides from *Streptococcus pneumoniae* and lipopolysaccharide from *Klebsiella pneumoniae* subsequently promoting recognition, phagocytosis, and destruction of the bacterial cell^10^. Additionally, although detailed mechanisms underlying the proteolytic cleavage of CD206 are not fully understood, the shedding of CD206 from the membrane is enhanced upon the recognition of several pathogens^18^. The present study showed elevated sCD206 concentrations in accordance with CAP severity and the accumulation of CD206-positive macrophages in the lungs of fatal patients with CAP. These results suggested that increased sCD206 levels represent the activation of CD206^+^ macrophages during this disease and might partly result from macrophage–pathogen interactions.

A dichotomous approach to macrophage polarization is essential to understand the immune response. M1 macrophages are essential for anti-microbicidal and anti-tumor responses, whereas M2 macrophages have a central role in tissue repair and the resolution of inflammation^19,20^. CD206 is considered a marker of the M2 phenotype^6–9^. Although the precise role of M2 macrophages in infectious disease with regard to complex M1/M2 polarization remains unknown, increased expression of CD206 was previously reported in monocytes from patients with sepsis^21,22^. Additionally, the deletion of CD206^+^ macrophages exacerbates lung injury in endotoxemic mice^23^ and during *Cryptococcus* infection^24^. CD206^−/−^ mice have been also reported to show increased allergic airway inflammation together with an elevated Th2/Th17 response^25^. This suggests a protective role for M2 macrophages and sCD206 by inhibiting excessive inflammation.

It is well established that PSI and CURB65 predict mortality of CAP patients with high sensitivity and specificity. Meanwhile, it has been reported that these indicators might not be accurate for elderly patients with CAP. As atypical clinical presentations are usually found in elderly patients with CAP, the cut-off values for the scoring system could prove unreliable in these patients^14–16^. Although comorbidities are more frequent and the relative risk of mortality is higher in elderly patients, there is also a risk of the misclassification of younger patients with severe disease into lower classes. We previously reported that modified PSI together with performance status can predict mortality more accurately than conventional PSI in patients with CAP ≥80 years of age^15^. Therefore, there is a need for age-independent surrogate markers to assess the severity of CAP. In this study, we demonstrated that sCD206 has the potential to predict mortality in patients with CAP, with a higher AUC value than that obtained for PSI. Additionally, sCD206 values were consistent in both age groups assessed, specifically, <75 years and ≥75 years, suggesting that this approach could complement PSI or CURB65 assessments.

To date, in line with our study on CD206, the utility of macrophage-related markers such as scavenger receptor CD163 and the immunomodulatory molecule IDO have been vigorously investigated in clinical settings with a variety of diseases, as a surrogate marker to estimate disease severity and/or prognosis^4,5,26–30^. In addition, elevated levels of sCD206 in patients with interstitial lung disease^31^, critical illness^32^, and pneumococcal bacteremia^33^ were also reported. Therefore, elevated levels of these molecules including sCD206 are not disease-specific but might also comprehensively represent a whole-body immune response.

The present study had several limitations. First of all, although we examined two independent cohorts of CAP patients, the sample size was still inadequate and univariate and multivariate analyses were not performed with each cohort separately, thus a further validation study is necessary. Therefore, the absolute significance of sCD206 in CAP could not be determined. Second, the results clearly indicate that increased sCD206 is observed in patients with CAP; however, the origin of sCD206- and CD206-expressing monocytes was not investigated. In addition, the role of increased sCD206 levels in the etiology of infectious diseases is not completely elucidated.

## Conclusion

In conclusion, the present study demonstrated elevated levels of serum sCD206 in patients with CAP, which is related to disease severity and clinical outcome. Furthermore, the sCD206 was useful for predicting outcome even in younger patients with CAP as well as elderly those with CAP. Our results suggested that sCD206 could be utilized as a novel prognostic marker for CAP and will help for better understanding the role of macrophage polarization in infectious disease.

## Methods

### Subjects

This prospective study was conducted using two cohorts of patients who were administered for the treatment of CAP in Hamamatsu, Japan. A cohort of 127 consecutive CAP patients admitted to Seirei-Mikatahara Hospital and Hamamatsu University School of Medicine between January 2007 and December 2010, and a cohort of 125 consecutive CAP patients hospitalized at Hamamatsu University School of Medicine between March 2013 and February 2018, were enrolled in this study. The former and latter cohorts were evaluated as a discovery cohort and validation cohort, respectively. Pneumonia was diagnosed according to previously published international guidelines^34,35^. CAP was defined as pneumonia that did not fulfill the criteria of hospital- or nursing and health care-acquired pneumonia in patients with symptoms of acute-onset lower respiratory tract infection, who demonstrated new infiltration on a chest radiograph. All patients were stratified into risk classes using the validated prediction rule, calculated according to the PSI and CURB-65 calculator (covering confusion, urea nitrogen, respiratory rate, and blood pressure, ≥65 years of age)^12,13^.

This study also included sera from 42 age and sex-matched subjects (30 men and 12 women, mean age of 73 years) who visited Hamamatsu University Hospital for health checks, as a control group. None of the control subjects had pulmonary infectious disease, as assessed by chest radiographs. This study was approved by the ethics committees of Hamamatsu University School of Medicine, Seirei-Mikatahara Hospital (15–167), and was carried out in accordance with approved guidelines. Written informed consent was obtained from all subjects in accordance with institutional guidelines. The study was registered in the University Hospital Medical Information Network in Japan (http://www.umin.ac.jp/. UMIN000003400 and UMIN000019472).

### Sample collection

Blood samples were drawn at the time of admission before the start of empirical treatment. Serum samples were frozen at −80 °C until analysis; routine laboratory examinations such as blood cell counts and biochemical analyses were subsequently performed. The serum concentration of sCD206 was determined using an enzyme-linked immunosorbent assay (ELISA) kit (Ray Biotech, Norcross, GA, USA).

### Immunohistochemistry

Lung biopsy specimens were obtained from autopsy cases, in which the patient died of severe pneumonia, and resected lung from a patient with early lung cancer was also examined. Tissues were fixed in 10% formalin and embedded in paraffin. Deparaffinized sections (5-μm-thick) were immersed in epitope retrieval solution (Target Retrieval Solution S1700; Dako North America, Inc., Carpinteria, CA, USA) and preheated at 120 °C for 10 min. After blocking endogenous peroxidase activity with 3% H_2_O_2_ for 15 min, slides were incubated overnight with a mouse anti-human CD206 monoclonal antibody (15 µg/ml; R&D Systems, Minneapolis, MN, USA) or IgG2b at 4 °C. Subsequently, sections were incubated with visualization reagent (ChemMate Envision kit; Dako Japan, Inc., Tokyo, Japan) for 30 min, followed by counterstaining with hematoxylin.

### Statistical analysis

Discrete variables are expressed as counts (percentage), and continuous variables are expressed as the median [range] unless otherwise specified. The Mann–Whitney test was used for continuous variables. Categorical data were compared between groups by using the Fisher’s exact test for independence. Correlations between sCD206 and clinical parameters were analyzed by the Spearman’s rank correlation method. Overall survival time was measured from the date of CAP diagnosis. To examine the ability of sCD206 levels to predict mortality in patients with CAP, combined cohort data were analyzed. The areas under the receiver operating characteristic (ROC) curve were used to evaluate the ability of this marker to predict mortality. The optimal cut-off value of sCD206 in the combined cohort that ensured the best combinations of sensitivity and specificity was obtained. Cumulative survival probabilities were estimated by the Kaplan–Meier method with optimal cut-off values. The Log-rank test was used to compare survival among patients. Univariate and multivariate analyses were performed with Cox proportional hazards regression analysis with combined cohort subjects to predict mortality. Among the statistically significant covariates in the univariate analyses, several were excluded because of potential confounders and statistical limitations. Statistical analyses were performed using GraphPad Prism Version 8 (GraphPad Software, San Diego, CA, USA) and EZR (Saitama Medical Center, Jichi Medical University, Saitama, Japan), which is a graphical user interface for R (The R Foundation for Statistical Computing, Vienna, Austria). Statistical significance was considered at a P-value of 0.05.

## Supplementary information

Supplementary information

## Data Availability

The data are available from the authors upon reasonable request.
