# Author Correction: Macrophage Mannose Receptor CD206 Predicts Prognosis in Community-acquired Pneumonia

**DOI:** 10.1038/s41598-020-58958-9

**Published:** 2020-02-19

**Authors:** Kazuo Tsuchiya, Yuzo Suzuki, Katsuhiro Yoshimura, Hideki Yasui, Masato Karayama, Hironao Hozumi, Kazuki Furuhashi, Noriyuki Enomoto, Tomoyuki Fujisawa, Yutaro Nakamura, Naoki Inui, Koushi Yokomura, Takafumi Suda

**Affiliations:** 1 Second Division, Department of Internal Medicine, Hamamatsu University School of Medicine, Hamamatsu, Japan; 2 Department of Respiratory Medicine, Seirei Mikatahara Hospital, Hamamatsu, Japan

Correction to: *Scientific Reports* 10.1038/s41598-019-55289-2, published online 10 December 2019

The original version of this Article contained a typographical error in the spelling of the author Kazuo Tsuchiya, which was incorrectly given as Tsuchiya Kazuo. This has now been corrected in the PDF and HTML versions of the Article, and in the accompanying Supplementary Information file.

