# Supplementary material for: Macrophage Mannose Receptor CD206 Predicts Prognosis in Community-acquired Pneumonia
Source: Sci Rep. 2019 Dec 10;9:18750. doi: 10.1038/s41598-019-55289-2 (PMC6904766; doi:10.1038/s41598-019-55289-2)
Supplement: Supplementary file 1 — Supplementary information [file 41598_2019_55289_MOESM1_ESM.pdf]

Macrophage Mannose Receptor CD206 Predicts Prognosis in Community-acquired Pneumonia

Tsuchiya Kazuo, M.D.[1*], Yuzo Suzuki, M.D., Ph.D.[1*#], Katsuhiro Yoshimura, M.D. [1], Hideki Yasui, M.D., Ph.D.[1], Masato Karayama, M.D., Ph.D.[1], Hironao Hozumi, M.D., Ph.D.[1], Kazuki Furuhashi, M.D., Ph.D.[1], Noriyuki Enomoto, M.D., Ph.D.[1], Tomoyuki Fujisawa, M.D., Ph.D.[1], Yutaro Nakamura, M.D., Ph.D.[1], Naoki Inui, M.D., Ph.D.[1], Koushi Yokomura, M.D., Ph.D.[2], Takafumi Suda, M.D., Ph.D[1]

[1]Second Division, Department of Internal Medicine, Hamamatsu University School of Medicine, Hamamatsu, Japan

[2] Department of Respiratory Medicine, Seirei Mikatahara Hospital, Hamamatsu, Japan

* These authors contributed equally to this work

[#]**Corresponding author:** Yuzo Suzuki, M.D., Ph.D.

Supplement Results

The following pathogens were detected: *Streptococcus pneumoniae* (n = 40; sputum culture = 31, blood culture = 10), *Staphylococcus aureus* (n = 18; sputum culture = 15, blood culture = 3), *Haemophilus influenzae* (n = 15; sputum culture), *Pseudomonas aeruginosa* (n = 11; sputum culture = 9, blood culture = 2), *Klebsiella pneumonia* (n = 5; sputum culture), *Mycoplasma pneumonia* (n = 2), *Legionella* spp. (n = 1), *Moraxella catarralis* (n = 1; sputum culture), *Escherichia coli* (n = 1; sputum culture), *Acinetobacter baumannii* (n = 1; sputum culture)*,* and an unidentified gram-negative bacillu*s* (n = 3; sputum culture) (Supplement Table 1).

Supplemental Fig 1. Immunohistochemical staining of CD206 in 69-year-old female autopsy case with community-acquired pneumonia.

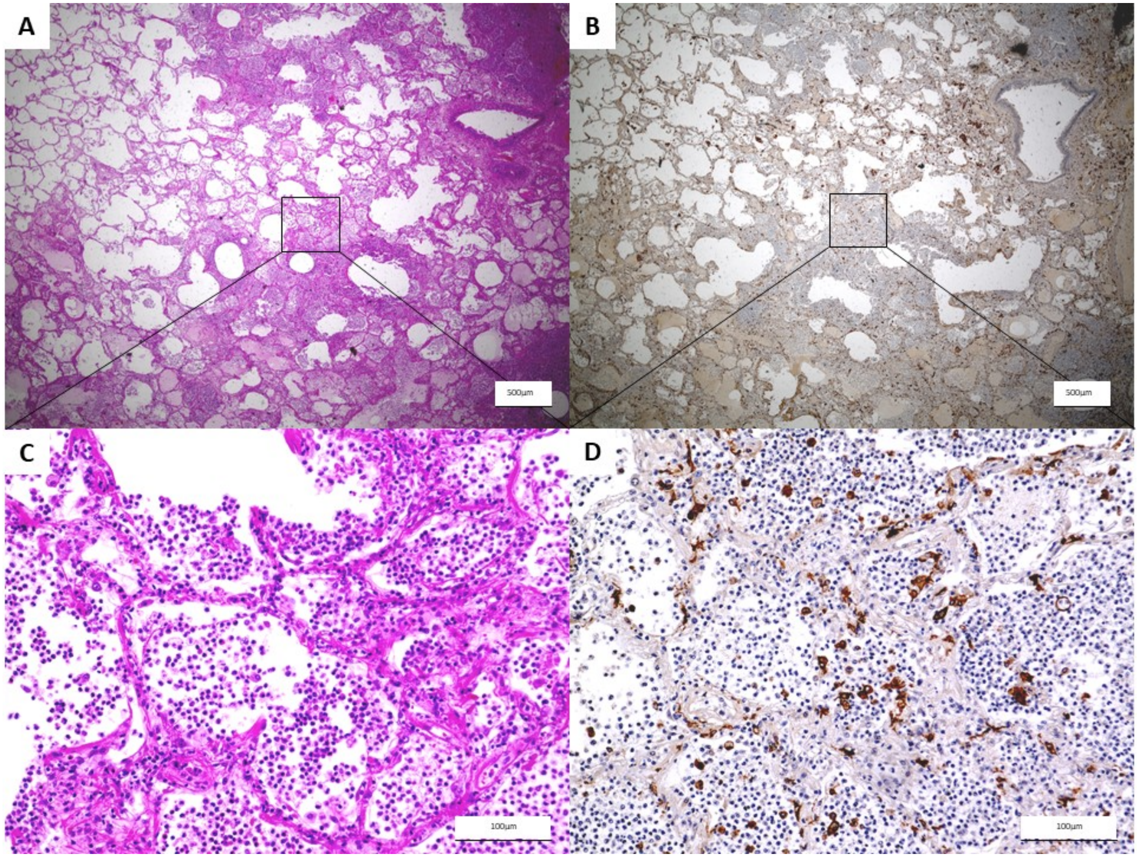

69-year-old female autopsy case, who was dead in two days from onset of pneumonia. (A:×20, C:×200) HE stain shows marked inflammatory cell infiltration and macrophages in alveolar. (B:×20, D:×200) CD206 positive macrophages were observed in immunohistochemical staining.

Supplemental Fig 2. Immunohistochemical staining of CD206 in 63-year-old male autopsy case with community-acquired pneumonia.

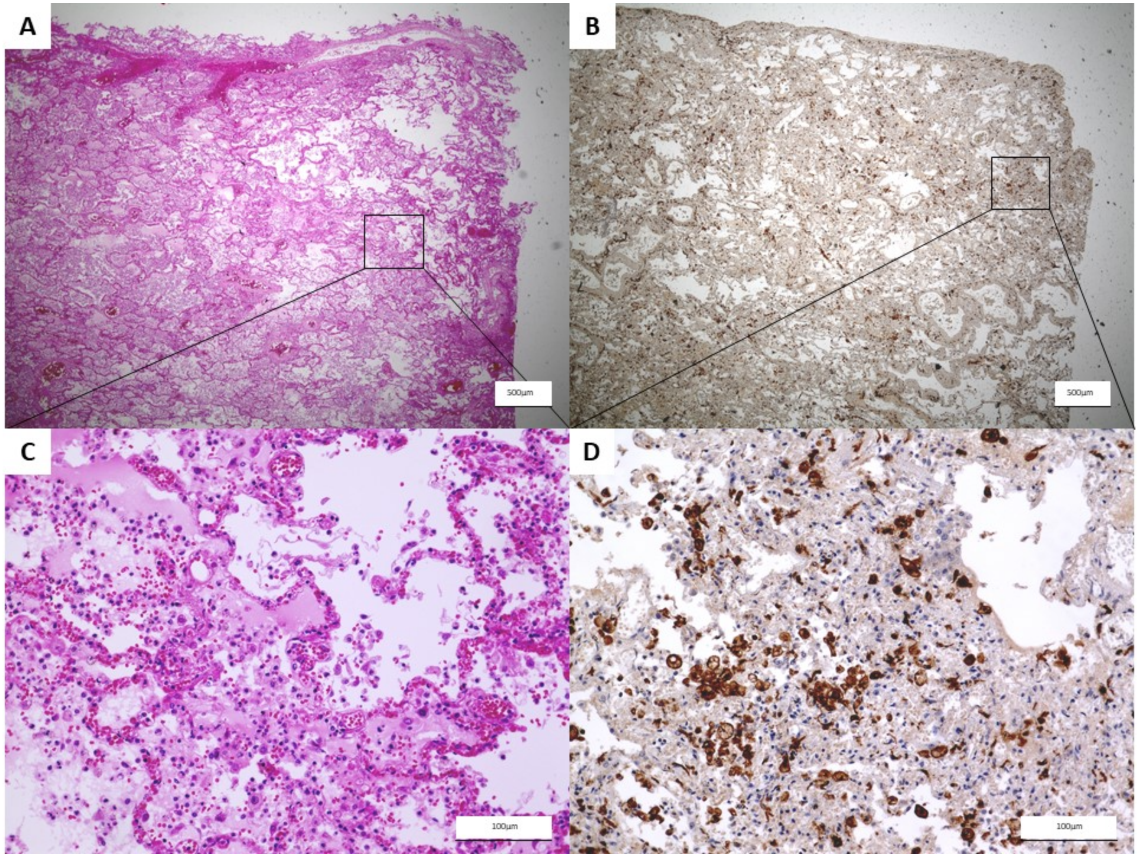

63-year-old male autopsy case, who was dead in five days from onset of pneumonia.

(A:×20, C:×200) HE stain shows macrophage infiltration, pulmonary congestion, and deposition of fibrin in the alveolar. (B:×20, D:×200)

CD206 positive macrophages were strongly observed in immunohistochemical staining.

Supplemental Fig 3. Immunohistochemical staining of CD206 in 55-year-old female case with early lung cancer.

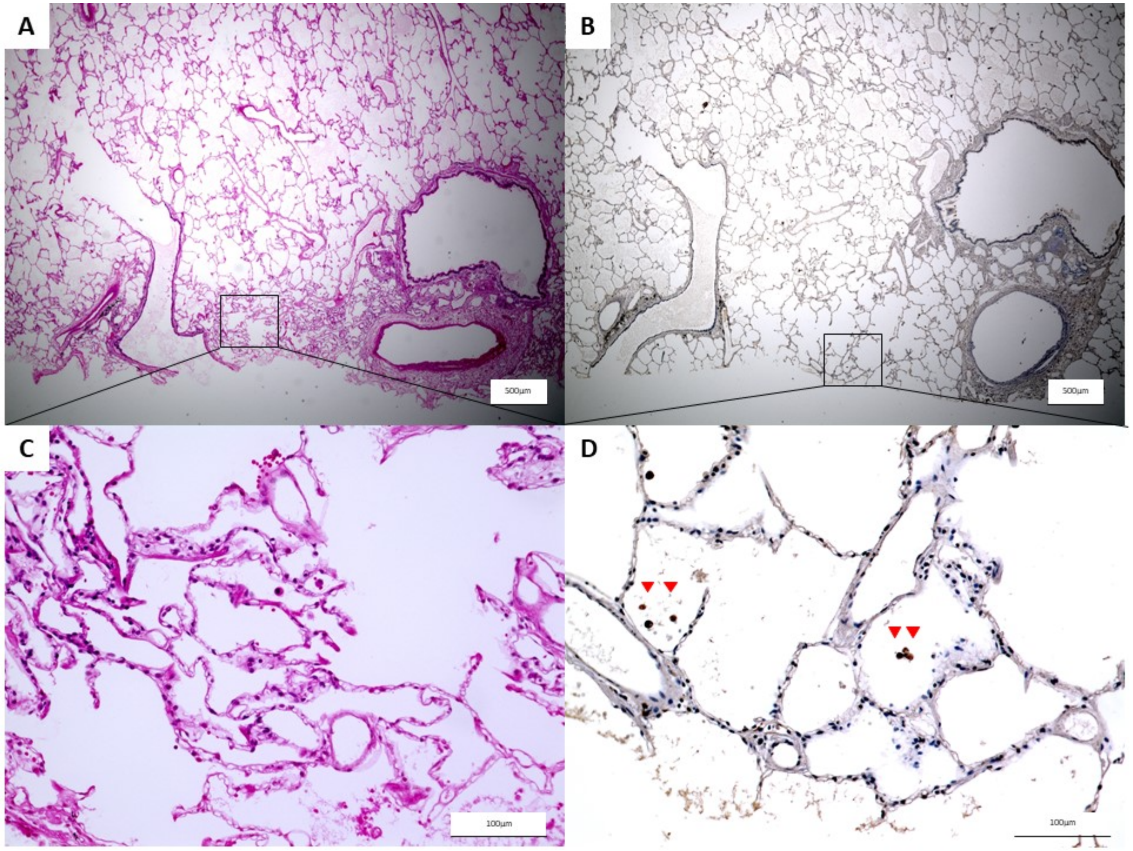

Lung section from a patient with early lung cancer, who was underwent partial resection.

(A:×20, C:×200) HE stain shows macrophages in the alveolar spaces. (B:×20, D:×200) CD206 positive macrophages (arrow head) were observed in immunohistochemical staining.

Supplemental Table1. Bacterial pathogen in community acquired pneumonia

	**Cohort1 (n=127)**	**Cohort2 (n=125)**	*p*-value
Streptococcus pneumoniae	24 (6)	16 (4)	0.228
Staphylococcus aureus	10 (1)	8 (2)	0.796
Hemophilus influenzae	7	8	0.808
Pseudomonas aeruginosa	5 (1)	6 (1)	0.768
Klebsiella pneumoniae	3	2	1.000
Mycoplasma pneumoniae	2	—	0.498
Legionella spp.	—	1	0.496
Moraxella catarrhalis	—	1	0.496
others	9	8	1.000
unknown (normal flora or not cultured)	68	75	0.312

Bacterial pathogens were detected from sputum culture and/or blood culture. The number shows positive sputum culture in the supplemental table1. The number in round bracket shows positive blood culture.

Supplemental Table2. Correlation between the soluble CD206 and clinical parameters in patients with community-acquired pneumonia

	**Cohort 1**		**Cohort 2**		**Cohort 1 + Cohort 2**	
	R	*p*-value	R	*p*-value	R	*p*-value
Age	0.3541	<0.001	0.06337	0.4827	0.2514	<0.0001
P/F ratio, mmHg	-0.1895	0.0425	-0.3253	0.0003	-0.3072	<0.0001
TP, g/dl	-0.1406	0.1178	-0.2540	0.0046	-0.2343	0.0002
Alb, g/dl	-0.3956	<0.0001	-0.4324	<0.0001	-0.4721	<0.0001
BUN, mg/dl	0.3164	0.0003	0.2327	0.0090	0.3055	<0.0001
Cre, mg/d	0.07897	0.3775	0.1325	0.1406	0.1730	0.0059
CRP, mg/dl	0.2401	0.0066	0.2304	0.0097	0.2412	0.0001
PCT, ng/ml	ND	ND	0.3159	0.0048	0.3159	0.0048
WBC, /µl	0.2320	0.0087	0.0906	0.3150	0.1482	0.0186
Ht, %	-0.2024	0.0225	-0.1564	0.0909	0.2036	0.0014
Plt, x 10^4/µl	0.0084	0.3506	-0.0537	0.5522	0.0146	0.8183
PSI (score)	0.4369	<0.0001	0.2802	0.0015	0.4035	<0.0001
CURB65 (score)	0.3386	<0.0001	0.3962	<0.0001	0.3516	<0.0001

P/F ratio; ratio of partial pressure arterial oxygen and fraction of inspired oxygen (PaO_2/FiO_2), TP; total protein, Alb; albumin, BUN; blood urea nitrogen, Cre; creatinine, CRP; C-reactive protein, PCT; procalcitonin, WB; white blood cell count, HB; hemoglobin, Ht; hematocrit, Plt; platelets, PSI; pneumonia severity index.

Supplemental Table3. **Receiver operator curve analysis for predicting mortality in patients with community-acquired pneumonia (CAP).**

Variable	AUC	Sensitivity (%)	Specificity (%)	Cut -off
WBC	0.542	27.3	89.6	17510
PCT	0.637	66.7	78.6	6.03
CRP	0.674	63.6	73.9	19.77
PSI	0.770	77.3	73.9	118
CURB65	0.747	77.3	57.0	1
CD206	0.856	86.4	79.1	1413

WBC, white blood cell count; PCT, procalcitonin; CRP, C-reactive protein; AUC, area under the curve

Supplemental Table4. **Receiver operator curve analysis for predicting mortality in each cohort.**

**Variable**	**Cohort1**				**Cohort2**			
	**Cut-off**	**AUC**	**Sensitivity (%)**	**Specificity (%)**	**Cut-off**	**AUC**	**Sensitivity (%)**	**Specificity (%)**
WBC	15700	0.539	38.5	81.7	10740	0.541	66.7	56.0
PCT	ND	ND	ND	ND	6.03	0.603	57.1	78.9
CRP	19.77	0.780	69.2	78.3	21.94	0.551	55.6	79.3
PSI	118	0.844	84.0	80.9	120	0.674	66.7	67.2
CURB65	3	0.744	38.5	95.7	1	0.773	88.9	55.2
CD206	1386.3	0.860	84.6	86.0	1868.5	0.876	77.8	87.9

WBC, white blood cell count; PCT, procalcitonin; CRP, C-reactive protein; AUC, area under the curve
